# Supplementary material for: Structural and Functional Characterization of the Holliday Junction Resolvase RuvC from Deinococcus radiodurans
Source: Microorganisms. 2022 Jun 6;10(6):1160. doi: 10.3390/microorganisms10061160 (PMC9228767; doi:10.3390/microorganisms10061160)
Supplement: Supplementary file 1 [file microorganisms-10-01160-s001.zip › microorganisms-1714905-supplementary.pdf]

Supplementary data

Table S1. The columns and buffers used for protein purification process.

**The component of the buffer used for each column**	
Nickel	Equivalent buffer: 500 mM NaCl, 30 mM Tris-HCl 7.5, 5% glycerol, 3 mM β-ME
	Elution buffer: 500 mM NaCl, 30 mM Tris-HCl 7.5, 5% glycerol, 3 mM β-ME, 300 mM imidazole
Desalting	200 mM NaCl, 30 mM Tris-HCl 7.5, 5% glycerol, 1 mM DTT
Heparin	Equivalent buffer: 200 mM NaCl, 30 mM Tris-HCl 7.5, 5% glycerol, 1 mM DTT
	Elution buffer: 1M NaCl, 30 mM Tris-HCl 7.5, 5% glycerol, 1 mM DTT
Superdex	200 mM KCl, 30 mM Tris-HCl 7.5, 5% glycerol, 1 mM DTT

Table S2. The columns and buffers used for protein purification process.

Oligos used for DNA binding and cleavage assays	
duplex	Sequence (5′- 3′)
duplex_1	CCACCAGAAACACGCCACAGTTTTTGTTTTGATTGCGAGGCCGTCCTACC
duplex_2	CCACCAGAAACACGCCACAGTTTTTGTTTTGATTGCGAGGCCGTCCTACC
splayed duplex	
splayed duplex_1	CCACCAGAAACACGCCACAGTTTTTGTTTTGATTGCGAGGCCGTCCTACC
splayed duplex_2	GGTAGGACGGCCTCGCAATCAAAACAAAAACTGTGGCGTGTTTCTGGTGG
bulge	
bulge_1	CCACCAGAAACACGCCACAGTTTTTGTTTTGATTGCGAGGCCGTCCTACC
bulge_2	GGTAGGACGGCCTCGCAATCTTTTGTTTTTCTGTGGCGTGTTTCTGGTGG
flap	
flap_1	CCACCAGAAACACGCCACAGTTTTTGTTTTGATTGCGAGGCCGTCCTACC
flap_2	GGTAGGACGGCCTCGCAATCAAAACTTTTTGAGCACGCGAGATGTCAACG
flap_3	CGTTGACATCTCGCGTGCTCAAAAA
Y-junction	
Y-junction_1	CCACCAGAAACACGCCACAGTTTTTGTTTTGATTGCGAGGCCGTCCTACC
Y-junction_2	GGTAGGACGGCCTCGCAATCAAAACTTTTTGAGCACGCGAGATGTCAACG
Y-junction_3	CGTTGACATCTCGCGTGCTCAAAAAAAAAACTGTGGCGTGTTTCTGGTGG
HJ-0X	
HJ-0X_1	CCACCAGAAACACGCCACAGTTTTTGTTTTGATTGCGAGGCCGTCCTACC
HJ-0X_2	GGTAGGACGGCCTCGCAATCAAAACTTTTTGAGCACGCGAGATGTCAACG
HJ-0X_3	CGTTGACATCTCGCGTGCTCAAAAAAAAAACAGATGCGGAGTGAAGTTCC
HJ-0X_4	GGAACTTCACTCCGCATCTGTTTTTAAAAACTGTGGCGTGTTTCTGGTGG
HJ-12X	
HJ-12X_1	GACGCTGCCGAATTCTGGCTTGCTAGGACATCTTTGCCCACGTTGACCCA
HJ-12X_2	TGGGTCAACGTGGGCAAAGATGTCCTAGCAATGTAATCGTCTATGACGTT
HJ-12X_3	AACGTCATAGACGATTACATTGCTAGGACATGCTGTCTAGAGACTATCGA
HJ-12X_4	TCGATAGTCTCTAGACAGCATGTCCTAGCAAGCCAGAATTCGGCAGCGTC

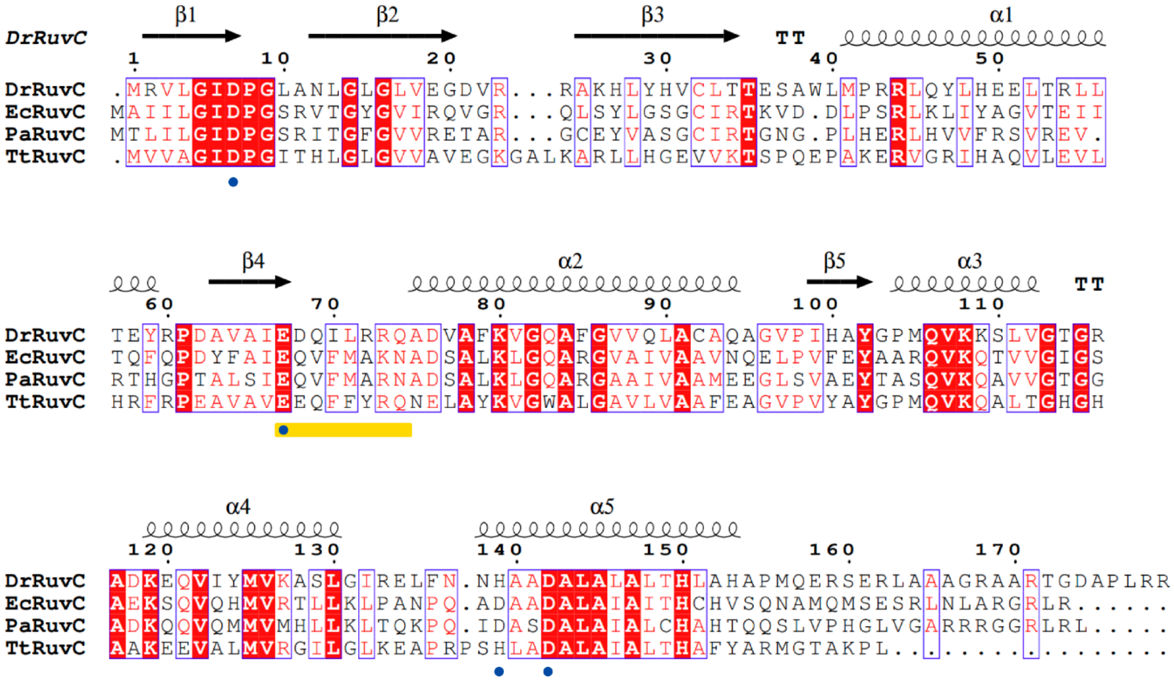

Figure S1. Multiple sequence alignment of RuvC homologs.

The secondary structure elements are indicated above the sequences, with the same labeling as Figure 3A. The numbers above the sequence indicate the residue numbers of *Dr*RuvC. The completely conserved amino acids are shaded in red. Blue circles indicate the amino acid residue of the catalytic active center. Yellow band indicates the base-specific recognition loop(BR-loop) that interacts with DNA. The alignment is generated with the online server of ESPript 3.0. Dr, *Deinococcus radiodurans*; *Ec, Escherichia coli*; *Pa, Pseudomonas aeruginosa*; *Tt, Thermus thermophilus*.

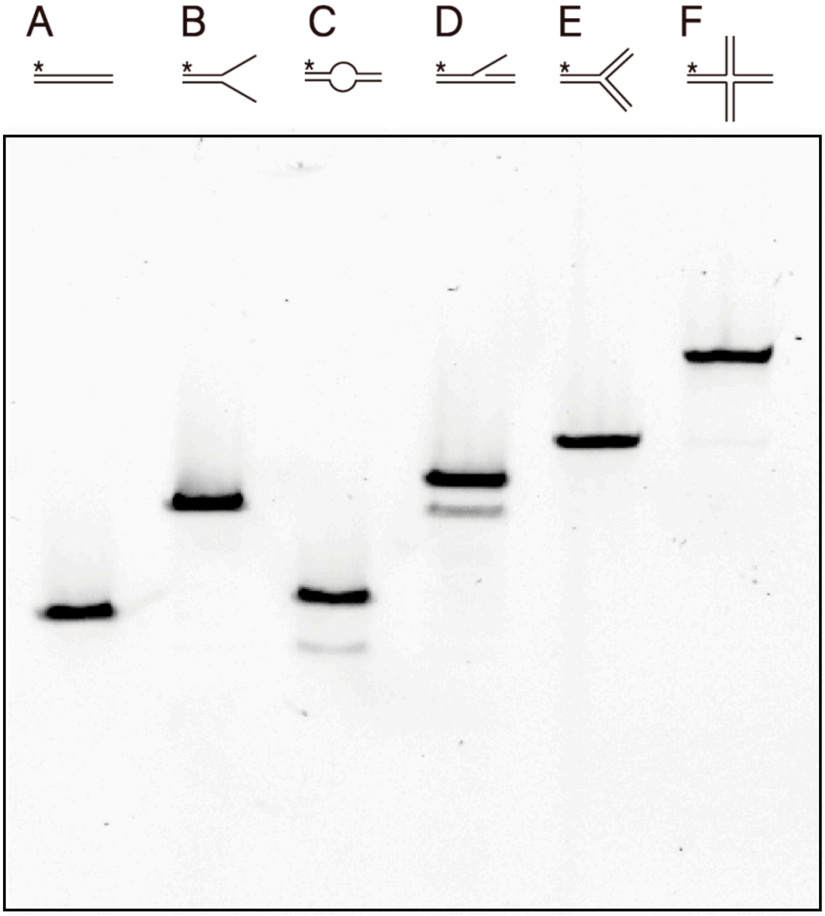

Figure S2. Annealing of DNA substrates used in this study.

A, duplex. B, splayed duplex. C, bulge. D, flap. E, Y -junction. F, Holliday junction.

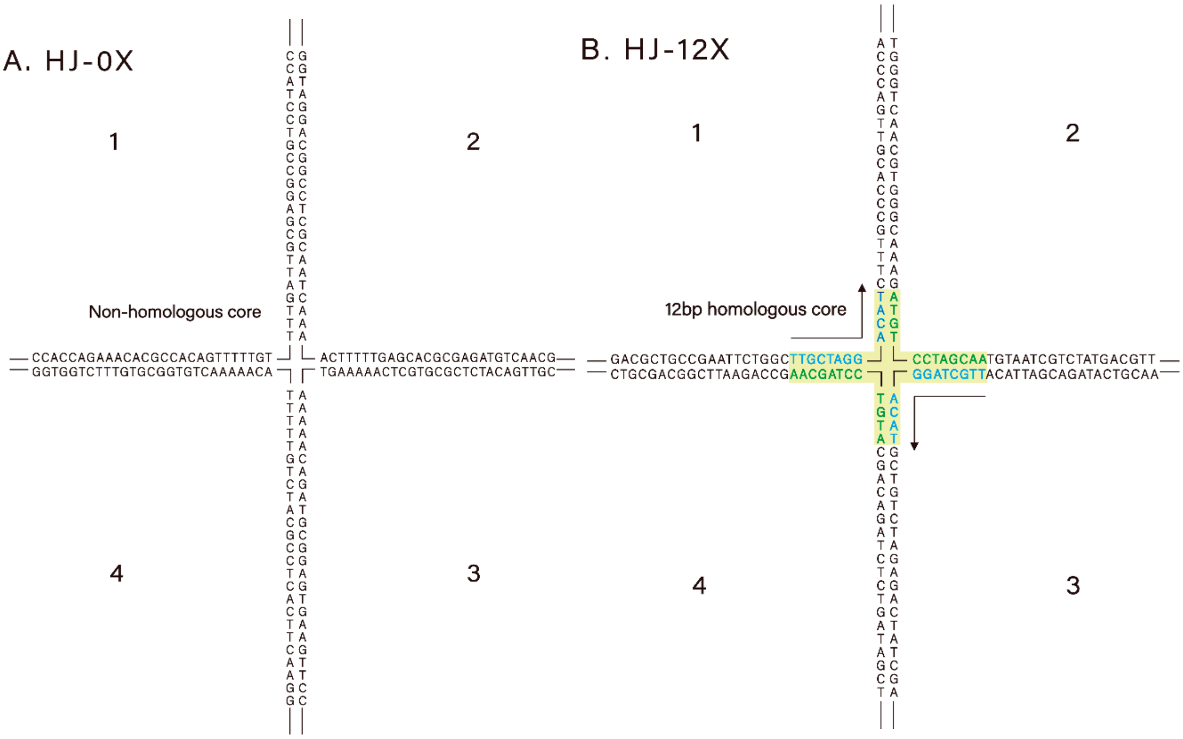

Figure S3. Two Holliday junction with different core.

(**A**) HJ-0X was prepared by annealing 4 DNA oligos with non-homologous core sequence (yellow). (**B**) HJ-12X was prepared by annealing 4 DNA oligos with 12 bp homologous core sequence (yellow).

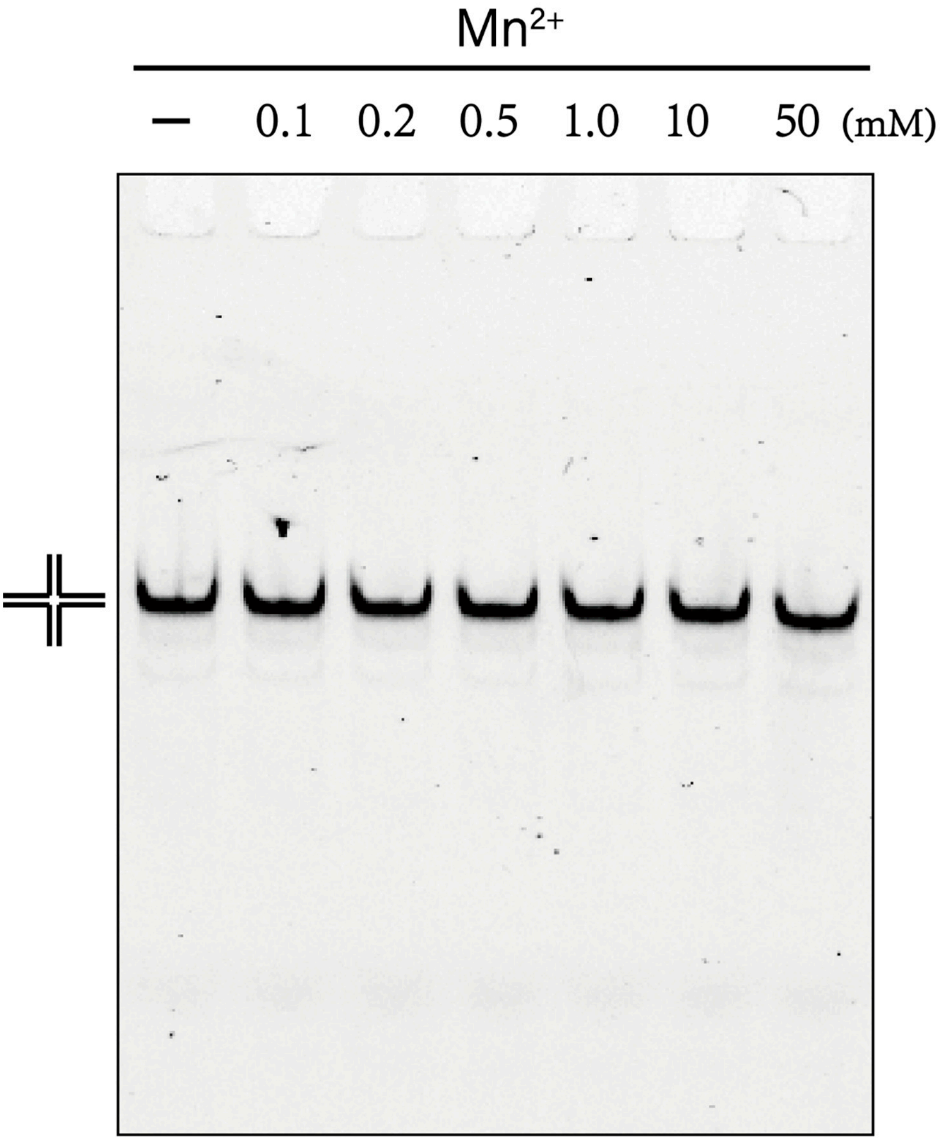

Figure S4. HJ-0X cleavage assays.

The HJ-0X cleavage assays were performed in the presence of 100 nM *Dr*RuvC and 50 nM HJs.

A

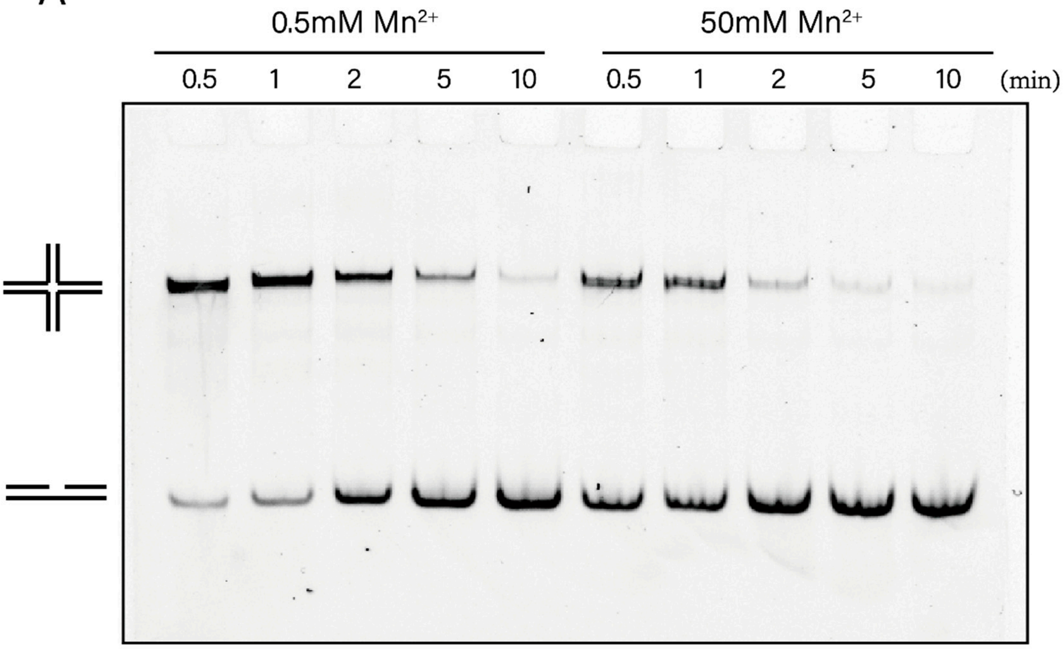

B

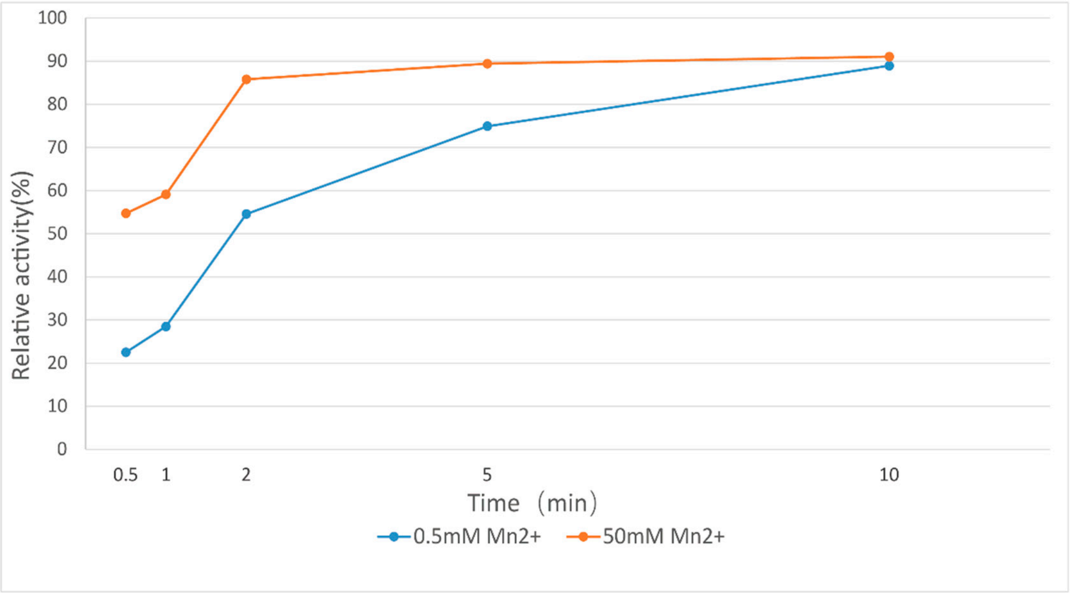

Figure S5. Time-resolved cleavage assay.

(**A**)The time-resolved cleavage assays were performed in the high or low concentration of Mn^{2+}. HJ-12X with 12bp homologous core was used as substrates. The cleavage assays were performed in the presence of 100 nM *Dr*RuvC and 50 nM HJs. (**B**) The combine time curve based on the bands density of the time-resolved cleavage assay.

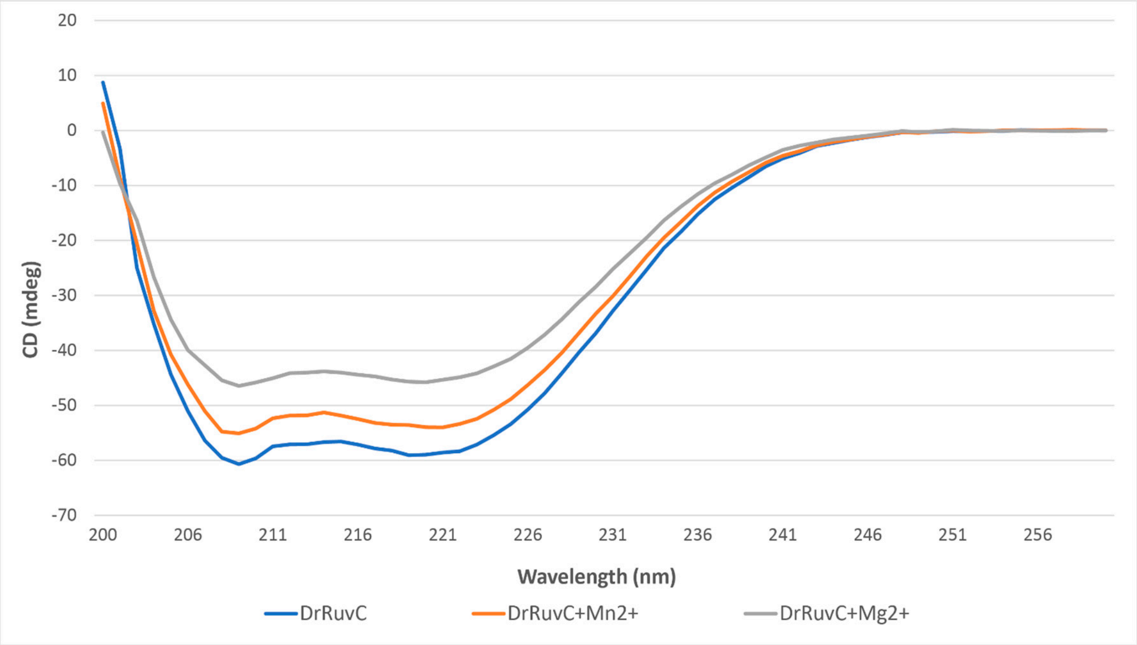

Figure S6. The CD results of *Dr*RuvC in different ion-containing buffer.

The configuration of *Dr*RuvC in different ion-containing buffer was determined by circular dichroism (CD) spectrum. The control is shown in blue in the absence of metal ions. The orange curve indicates *Dr*RuvC in the presence of 1mM Mn^{2+}. The grey curve indicates *Dr*RuvC in the presence of 1mM Mg^{2+}.
